# Shotgun metagenomic analysis of metabolic diversity and microbial community structure in experimental vernal pools subjected to nitrate pulse

**DOI:** 10.1186/1471-2180-13-78

**Published:** 2013-04-10

**Authors:** Sarah R Carrino-Kyker, Kurt A Smemo, David J Burke

**Affiliations:** 1 The Holden Arboretum, Kirtland, OH, USA; 2 Department of Biology, Case Western Reserve University, Cleveland, OH, USA; 3 Department of Biological Sciences, Kent State University, Kent, OH, USA

**Keywords:** Nitrate, Metagenome, 454 Sequencing, Environmental gene tag, Microcosm

## Abstract

**Background:**

Human activities have greatly increased nitrogen (N) levels in natural habitats through atmospheric N deposition and nutrient leaching, which can have large effects on N cycling and other ecosystem processes. Because of the significant role microorganisms play in N cycling, high inputs of nitrogenous compounds, such as nitrate (NO_3_-), into natural ecosystems could have cascading effects on microbial community structure and the metabolic processes that microbes perform. To investigate the multiple effects of NO_3_- pollution on microbial communities, we created two shotgun metagenomes from vernal pool microcosms that were either enriched with a solution of 10 mg NO_3_--N (+NO_3_-) or received distilled water as a control (−N).

**Results:**

After only 20 hours of exposure to NO_3_-, the initial microbial community had shifted toward one containing a higher proportional abundance of stress tolerance and fermentation environmental gene tags (EGTs). Surprisingly, we found no changes to N metabolism EGTs, even though large shifts in denitrification rates were seen between the +NO_3_- and –N microcosms. Thus, in the absence of NO_3_- addition, it is plausible that the microbes used other respiratory pathways for energy. Respiratory pathways involving iron may have been particularly important in our –N microcosms, since iron acquisition EGTs were proportionally higher in the –N metagenome. Additionally, we noted a proportional increase in *Acidobacteria* and *Alphaproteobacteria* EGTs in response to NO_3_- addition. These community shifts in were not evident with TRFLP, suggesting that metagenomic analyses may detect fine-scale changes not possible with community profiling techniques.

**Conclusions:**

Our results suggest that the vernal pool microbial communities profiled here may rely on their metabolic plasticity for growth and survival when certain resources are limiting. The creation of these metagenomes also highlights how little is known about the effects of NO_3_- pollution on microbial communities, and the relationship between community stability and function in response to disturbance.

## Background

Human activities, particularly agricultural practices and fossil fuel emissions, have greatly increased inputs of nitrogen (N) to terrestrial and aquatic habitats [1]. In agricultural regions, N is leached from soil in the form of nitrate (NO_3_-), which is often found in high concentrations in groundwater and groundwater-fed surface waters [2,3]. Moreover, high NO_3_- in surface runoff is often observed when fertilizer is used [4,5]. These sources of NO_3_- pollution pose a particular threat to aquatic habitats where groundwater and surface runoff are a significant or primary source of input. Vernal pools are temporary aquatic habitats that are common to temperate regions and filled by surface runoff following snowmelt, spring rain, and rising water table [6]. As such, N enrichment from NO_3_- leaching can alleviate N limitation and have a significant influence on N cycling. Because vernal pools are shallow depressions that often experience low dissolved oxygen concentrations [7-9], increased NO_3_- availability can favor anaerobic N cycling processes, such as denitrification and anaerobic ammonium oxidation, while suppressing anoxic pathways adapted to low NO_3_- conditions, such as dissimilatory nitrate reduction to ammonium.

N cycling is almost exclusively mediated by microorganisms; therefore high NO_3_- inputs can influence N cycling and also have cascading structural effects on the microbial communities involved. By studying genes for the enzymes responsible for the conversion of N between oxidized and reduced forms, there have been large advances in our knowledge of microbial functional groups involved in N cycling [10,11]. However, the N cycle is a complex network of pathways that can share some enzymes and can also be simultaneously influenced by the input of one nitrogenous compound, such as NO_3_- [12]. Therefore, studies which profile only one or a subset of N cycling enzymes may provide a limited view of how NO_3_- pollution impacts microbial processes. In addition, most previous studies on the effects of NO_3_- on microbial functional genes have limited their assessment to N cycling genes (e.g., [13,14]), even though elevated NO_3_- is known to affect other microbial processes, such as those involved in C cycling (e.g., [15,16]). One method that could help overcome these limitations is a shotgun metagenomic approach, where multiple functional genes can be examined.

In this study, we utilized a shotgun metagenomic approach to examine the multiple effects of NO_3_- addition on vernal pool microbial communities in a microcosm experiment [17]. Two metagenomes were created, one for replicate microcosms that received NO_3_- (labeled +NO_3_-) and one for replicate microcosms where NO_3_- was not added (labeled –N). Our previous study using these microcosms found that the addition of NO_3_- increased denitrification, while denitrification was not detected in the absence of NO_3_- [17]. This functional change was not accompanied by any change in the denitrifier community structure, which was profiled with the *nosZ* gene using terminal restriction fragment length polymorphism (TRFLP) [17]. It is unclear, however, if this lack of response by the denitrifying community was physiological in nature or related to our functional gene choice. For the shotgun metagenomic method utilized here, the microbial genomes were randomly amplified, thus allowing for the potential inclusion of multiple N cycling genes, as well as genes involved in other microbial processes. In addition to denitrifier community structure, our previous analyses used TRFLP to profile the structure of general bacteria and fungi, which also did not respond to NO_3_- addition [17]. Because shotgun metagenomes also provide taxonomic information for microbial communities, we hypothesized that inclusion of more than one functional gene and obtaining taxonomic composition using a shotgun metagenomic approach would reveal community structural responses to NO_3_- pulses not observed with the profiling technique, TRFLP.

## Results

For the +NO_3_- metagenome, there were 28,688 DNA fragments for a total of 9,085,193 bp and an average sequence length of 316 bp. The –N metagenome contained a larger number of DNA fragments with 81,300 and a total sequence length of 30,630,623 bp with an average fragment size of 376 bp. The metagenomes were uploaded to the Meta Genome Rapid Annotation of Sequence Technology (MG-RAST) server [18] and were analyzed unassembled with a BLASTX comparison to the SEED subsystems [19], which provided both taxonomic composition and metabolic functions. After applying our filters of 10^-5^ or lower e-value and 50 bp or greater sequence similarity, 7,406 sequences (+NO_3_-) and 14,063 sequences (−N) from the metagenomes matched with subsystems following the BLASTX analysis. The number of sequence matches to taxa with the BLASTX comparison were 6,342 (+NO_3_-) and 12,241 (−N). Each of these characterized DNA fragments represented an environmental gene tag (EGT), or a short segment of a gene found in the microcosm samples. The MG-RAST output included metabolic functions at four different levels, with subsystem category as the highest level and a specific gene as the lowest (see Table 1 for an example). The taxonomic output included EGT matches to domain, phylum, class, order, family, genus, and species; however because of the low sequence size cutoff of 50 bp, class was the lowest taxonomic group analyzed.

**Table 1:** Environmental gene tag (EGT) matches to lower levels in the SEED database that were significantly different with Fisher exact tests

	**EGT match**			**Proportional representation (%)**	
**Subsystem category**^**1**^	**Level 2**	**Level 3**	**Function**	**+NO**_**3**_**-**	**--N**
Fatty acids, lipids, and isoprenoids	Phospholipids	Glycerolipid and Glycerophospholipid Metabolism in Bacteria	Aldehyde dehydrogenase	0.85	0
Fatty acids, lipids, and isoprenoids	Isoprenoids			1.04	0.49
Iron acquisition and metabolism	Iron acquisition in *Vibrio*	-	TonB-dependent receptor	0	0.75
Stress response	Oxidative Stress	Oxidative stress	Alkyl hydroperoxide reductase subunit C-like protein	1.22	0.17
RNA metabolism	RNA processing and modification			1.66	2.70
Carbohydrates	CO_2_ fixation	Calvin-Benson cycle	NAD-dependent glyceraldehyde-3-phosphate dehydrogenase	1.55	0.25
Carbohydrates	Fermentation	Acetyl-CoA fermentation to Butyrate		1.88	1.24
Protein metabolism	Protein processing and modification	G3E family of P-loop GTPases (metallocenter biosynthesis)	Urease beta subunit	0	0.82

^1^ The lowest significant level for each category is reported here. Only the subsystem categories that were significantly different with Fisher exact tests (see Figure 2) are reported here. See Additional file 1: Tables S1-S3 for complete results of Fisher exact tests.

**Figure 1:**
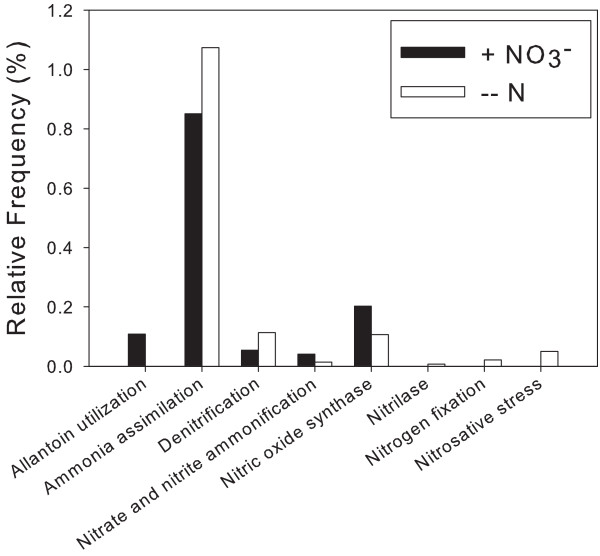
**Subsystem matches in the nitrogen metabolism category.** The proportional numbers of environmental gene tags that matched with level 2 sequences within the nitrogen metabolism subsystem category for the +NO_3_- (solid bars) and –N (open bars) metagenomes. No significant differences were found when these sequences were analyzed with Fisher exact tests in the Statistical Analysis of Metagenomic Profiles program.

Although NO_3_- addition increased denitrification rate (mean = 3.84 ± 0.44 mg N (kg soil)^-1^ day^-1^ versus not detected in the microcosms receiving distilled water), no significant differences in nitrogen metabolism EGTs were found with the BLASTX comparison to the SEED database (Figure 1). Results from Fisher exact tests at all subsystem levels and a chi-square test conducted at level two indicated no statistical differences between the N metabolism EGTs (Additional file 1: Tables S1-S4). Of the 7,406 EGT matches to the SEED database in the +NO_3_- metagenome, only 93 (1.26%) were to nitrogen metabolism subsystems. Likewise, a low percentage of SEED database EGT matches (195 of 14,063 EGT matches; 1.39%) were to nitrogen metabolism subsystems for the –N metagenome. Additional analysis of N metabolism EGTs was conducted with a BLASTN comparison of the metagenomes to a database of genes involved in N cycling pathways that we created from searches at the NCBI site. The database included genes for the enzymes involved in denitrification, dissimilatory nitrate reduction to ammonium (DNRA), anaerobic ammonium oxidation (Annamox), nitrification, and N fixation. (A complete list of the genes included in the database can be found in Additional file 2: Table S5). Only the +NO_3_- metagenome contained matches to the N metabolism database with the BLASTN, which included two sequences (out of 28,688 or 0.0070%) from the +NO_3_- metagenome that matched with a number of nitrate reductase sequences (Table 2 and Additional file 2: Table S6). (All other matches in the +NO_3_- metagenome and all matches to the –N metagenome were to genes not involved in N metabolism, but were picked up by our NCBI searches because they were chromosomes that also included a N metabolism gene that was searched for.)

**Table 2:** Nitrogen metabolism gene matches and the number of sequences from the +NO_3_- metagenome that matched with the genes, as determined with a BLASTN comparison

**Query sequence**^1^	**N Metabolism gene**	**# Database sequences**	**Average%ID**	**Average alignment length**	**Average E-value**
+NO_3_- seq. 1	*napA*	3	92.83	65	7.33E-18
+NO_3_- seq. 2	*napA*	125	83.83	131.29	9.86E-08
	*napB*	1	82.35	119	4.00E-11

^1^The query sequence indicates that only two sequences out of 28,688 in the +NO_3_- metagenome matched with sequences in the N metabolism database. Seq. 1 matched with three database entries, while seq. 2 matched with 126 database entries.

**Figure 2:**
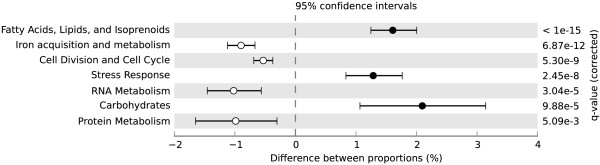
**Significant subsystem differences between the +NO_3_- and –N metagenomes.** Results of a Fisher exact test (conducted with the Statistical Analysis of Metagenomic Profiles program) showing the significant differences of subsystem environmental gene tag (EGT) matches between treatments. Higher EGT relative abundance in the +NO_3_- metagenome have a positive difference between proportions (closed circles), while higher EGT relative abundance in the –N metagenome have a negative difference between proportions (open circles).

EGT matches to other subsystems found with the BLASTX comparison to the SEED database, however, changed significantly between the treatments (Figure 2, Table 1, and Additional file 1: Tables S1-S4). EGTs that matched with genes in the categories of iron acquisition and metabolism, cell division and cell cycle, RNA metabolism, and protein metabolism were proportionally higher in the –N metagenome (Figure 2). The +NO_3_- metagenome contained a higher relative number of EGT matches to genes in the fatty acids, lipids, and isoprenoids, stress response, and carbohydrates categories (Figure 2). Lower level metabolic EGT matches within these categories that were significantly different between the metagenomes are listed in Table 1.

**Figure 3:**
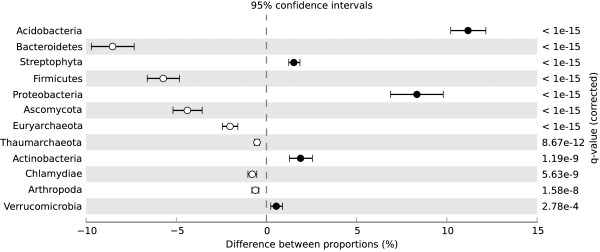
**Significant phylum differences between the +NO_3_- and –N metagenomes.** Results of a Fisher exact test (conducted with the Statistical Analysis of Metagenomic Profiles program) showing the significant differences of environmental gene tag (EGT) matches to phyla between treatments. Higher EGT relative abundance in the +NO_3_- metagenome have a positive difference between proportions (closed circles), while higher EGT relative abundance in the –N metagenome have a negative difference between proportions (open circles).

**Figure 4:**
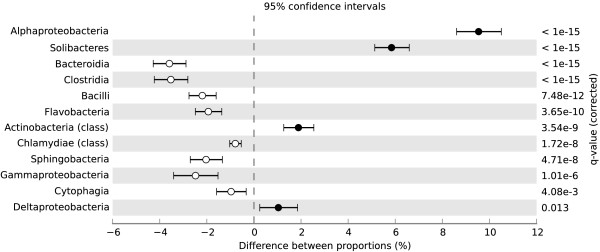
**Significant class differences in the domain bacteria between the +NO_3_- and –N metagenomes.** Results of a Fisher exact test (conducted with the Statistical Analysis of Metagenomic Profiles program) showing the significant differences of environmental gene tag (EGT) matches to class between treatments. Higher EGT relative abundance in the +NO_3_- metagenome have a positive difference between proportions (closed circles), while higher EGT relative abundance in the –N metagenome have a negative difference between proportions (open circles).

At the phylum level, EGT matches to *Acidobacteria*, *Proteobacteria*, *Actinobacteria*, and *Virrucomicrobia* in the domain *Bacteria* and *Streptophyta* in the domain *Eukaryota* were proportionally higher in the +NO_3_- metagenome (Figure 3). EGT matches to the phyla *Bacteroidetes*, *Firmicutes*, and *Chlamydiae* in the domain *Bacteria*, to *Euryarchaeota* and *Thaumarchaeota* in the domain *Archaea*, and to *Ascomycota* and *Arthropoda* in the domain *Eukaryota* were proportionally higher in the –N metagenome (Figure 3). Significant differences between the metagenome taxa were also deduced at the class level to specifically examine differences within the *Proteobacteria* phylum (Figure 4). EGT matches to *Alphaproteobacteria* and *Deltaproteobacteria* were proportionally higher in the +NO_3_- metagenome, while matches to *Gammaproteobacteria* were relatively higher in the –N metagenome (Figure 4).

## Discussion

Metagenomic analysis revealed treatment differences both for functional and taxanomic EGTs between our +NO_3_- and –N metagenomes. These differences were apparent even though the metagenome sequencing conducted here returned a lower number of sequences than are typically reported for shotgun metagenome studies [20-22]. However, a shotgun metagenomic sequencing effort conducted by Fierer et al. [23], where comparable sequence numbers to ours are reported, was able to elucidate increases in functional genes with increased N fertilization, suggesting that our sequence numbers are adequate for determining relative metabolic and taxonomic changes.

A somewhat surprising result was no proportional abundance change in any of the N metabolism EGTs between our treatments with the BLASTX comparison to the SEED database. Particularly surprising was no change in the denitrification EGTs (determined with the BLASTX) between treatments and no detection of denitrification genes with the BLASTN, other than two sequence matches to nitrate reductase in the +NO_3_- treatment. The two sequence matches with the BLASTN in the +NO_3_- metagenome were to the nitrate reductase genes *napA* and *napB*. Because the periplasmic nitrate reductases, which are the products of *napA* and *napB*, are used in both denitrification and DNRA [12], no conclusions can be drawn on which of these microbial groups grew to a level where they could be detected in the +NO_3_- microcosms. This lack of EGT response was despite the fact that we observed denitrification rate responses to our treatments [17], where the microcosms receiving NO_3_- displayed a denitrification rate near or higher than the upper range of what has been measured in flooded soils in the field [24]. This result is consistent with a number of other studies that have found no link between function (including measurements of denitrification rate and denitrifying enzyme activity) and denitrifier gene copy number using QPCR [13,25-27]. We previously suggested that, in the absence of NO_3_- addition, denitrifiers in our microcosms used other electron acceptors for respiration when NO_3_- was not available [17], since denitrifiers are known to use other respiratory pathways [see review 10]. There were proportionally higher EGTs in the iron acquisition and metabolism category in the –N metagenome, and the specific EGT match was to a TonB-dependent receptor (Table 1). TonB-dependent receptors are a category of energy-coupling proteins, which are known to be involved in iron uptake by members of the genus *Pseudomonas*[28,29], and there is some evidence that one specific TonB-dependent receptor is involved in dissimilatory iron reduction by *Shewanella oneidensis*[30]. This suggests that the microbial community in the –N microcosms contained a greater number of organisms capable of acquiring iron and, perhaps, utilizing it for energy, which may have been a potential survival strategy in the absence of the NO_3_- addition. To our knowledge, evidence to support this hypothesis is sparse (but see Hauck et al. [31], who found that denitrifiers can also perform anaerobic ferrous iron oxidation). It is accepted, however, that denitrifying organisms primarily perform aerobic respiration and then switch to denitrification under anoxic conditions where NO_3_- supply is sufficient [32]. There is a category available through MG-RAST for respiration genes. There were close to 400 EGT matches from the two metagenomes to this category for genes involved in both aerobic and anaerobic respiratory pathways. However, there were no proportional changes in respiration EGT abundance between the +NO_3_- and the –N conditions (data not shown), likely because the microcosms were made anoxic prior to the metagenome creation, which could negate any advantage to aerobic organisms in either treatment. Though we did not observe proportional changes for EGTs involved in a known alternative respiratory pathway for denitrifiers, the observed proportional increase in iron acquisition and metabolism EGTs in the –N metagenome suggests that iron might be biogeochemically important under anoxic N-limited conditions.

Another possible reason for lack of denitrifier EGT treatment response is that denitrifiers may have been in low abundance compared to other microbial groups, making changes to their population undetectable relative to the background population numbers. For example, the denitrification gene *nosZ* is known to be in low abundance compared to 16S genes [33], and there are estimates that only 0.1 – 5% of culturable soil bacterial species can carry out denitrification [34]. This conclusion is supported by our BLASTN results, which found only two sequences from either metagenome that matched with a N metabolism gene. With the BLASTX comparison to the SEED database, however, over 1% of our sequences from each metagenome matched with nitrogen metabolism subsystems. The fact that we found no differences in nitrogen metabolism EGT relative abundance after NO_3_- addition suggests that microbial populations involved in N cycling did not shift in the 20 hours following exposure to a NO_3_- pulse. This lack of treatment response could be due to insufficient time between treatment initiation and sampling (i.e. populations were slow to respond to the treatment). However, we did see other EGT changes, suggesting that some microbial populations grew and experienced a detectable community shift in response to acute changes in NO_3_- concentration. The initial microbial community response to NO_3_- in our metagenomes was toward organisms that contained stress response, carbohydrate, and fatty acids, lipids, and isoprenoid EGT matches (Figure 1). The stress response EGT that was higher in the +NO_3_- metagenome was for an alkyl hydroperoxide reductase subunit C-like protein. The gene for alkyl hydroperoxide reducates, subunit C is upregulated by NO_3_- exposure after only 30 minutes in *Desulfovibrio vulgaris*, suggesting that such increases in this and other oxidative stress genes may be a general stress response by the bacteria [35]. Within the carbohydrates category, one EGT match that was higher in the +NO_3_- metagenome was for fermentation. Recently, there has been evidence for fermentation that is coupled to NO_3_- reduction in both bacteria and fungi [36,37]. Fermentation in the +NO_3_- microcosms may have been particularly prominent for the fungi, because a switch to NO_3_- -coupled fermentation as the primary source of energy for soil fungi under anoxic conditions has been suggested [36].

The sequencing effort described here also showed changes to the proportional representation of taxonomic EGTs. There were highly significant increases in the relative abundance of *Alphaproteobacteria* and *Acidobacteria* EGTs in the +NO_3_- metagenome. Similarly, using freshwater microcosms, Barlett and Leff [38] found an increase in *Alphaproteobacteria* abundance when NO_3_- was present as a N source and suggested a competitive advantage to this group of organisms under these conditions. Under anoxic conditions, such as our microcosms, higher physiological activity and substrate uptake have been reported in several *Alphaproteobacteria* species when NO_3_- or NO_2_- were present as an electron acceptor [39]. Therefore, in our microcosms, there could have been a competitive advantage to the *Alphaproteobacteria* due to greater growth compared to other facultative organisms in an anoxic environment with abundant NO_3_-. To our knowledge, there have been no other studies that found such an increase in *Acidobacteria* with NO_3_- addition. However, a sequencing effort in cultured strains of *Acidobacteria* recently found that these organisms possess NO_3_- and NO_2_- reducing genes [40]. *Alphaproteobacteria*[41], and likely *Acidobacteria*[40], are adapted to low nutrient conditions. While this seems counterintuitive to our microcosm study, vernal pools in nature are known to be oligotrophic [7]. The *Alphaproteobacteria* and *Acidobacteria* in vernal pools, then, may be adapted to survival in the disturbed, low nutrient conditions of these habitats and once NO_3_- becomes readily available they have a competitive advantage due to their growth capabilities in the presence of NO_3_-.

These taxonomic changes were not found in a previous examination of general bacteria or general fungi in these microcosms with TRFLP [17]. The metagenomic analysis reported here provides a greater resolution than TRFLP, which is a coarse community profiling tool. Therefore, there may have been fine-scale changes in bacterial community structure that were not detected with TRFLP. Another reason for this discrepancy is that our previous TRFLP analyses used the gene regions of bacterial 16S and fungal ITS for profiling [17] and, in the current study, a nonredundant protein database was used for taxonomic comparisons. Therefore, the conclusions drawn here regarding taxonomic changes may be limited to the taxonomic groups that changed functionally. The fact that whole genome amplification (WGA) was used prior to 454 sequencing could also be contributing to the differences seen between the metagenomes that were not noted with TRFLP. This is because amplification techniques with the Phi29 DNA polymerase, which was used in the current study, have been shown to exclude the amplification of certain DNA sequences, particularly those in low abundance or those that are GC rich, and can skew the representation of certain OTUs compared to sequencing efforts of non-amplified DNA of the same sample [42-44]. Additionally, our study design cannot exclude the possibility that the communities changed between the treatments over the 30 day incubation period prior to our sample collection. Thus, differences seen between the metagenomes may not be only because of the NO_3_- addition, but could also be due to an incubation period that changed the communities in the separate microcosms. There were six replicate microcosms to help control for variability between each jar, and our previous TRFLP profiling of the bacterial and fungal communities and the *nosZ* gene showed no differences in community structure between the +NO_3_- and –N microcosms [17]. Therefore, we expect community changes in response to the 30 day incubation to be minimal compared to the NO_3_- addition. Nevertheless, the observed proportional increase in *Alphaproteobacteria* and *Acidobacteria* in response to NO_3_- addition in the metagenomes requires more in depth study on the ecology of these groups and how they tolerate NO_3_- pollution.

## Conclusions

These results suggest that NO_3_- additions to vernal pool habitats may be accompanied by relatively rapid microbial community changes at both the functional and taxonomic level. The initial community shift after only 20 hours of NO_3_- exposure was toward a more stress tolerant community capable of performing fermentation and away from a community more dependant on respiratory pathways involving iron, as evidenced by higher iron acquisition EGTs in the –N microcosms. Surprisingly, we found no changes to N metabolism EGTs with the BLASTX in response to our treatments and only a two sequence increase in detection of nitrate reductase genes, despite a vast increase in denitrification rate with NO_3_- addition. Thus, in the absence of an NO_3_- addition, it is plausible that denitrifying microbes used other respiratory pathways for energy and, although NO_3_- addition altered their metabolic response, it did not alter or affect community structure or size. Because microbial communities are diverse, they are thought to be functionally redundant [45-47]. Our results suggest that the vernal pool microbial communities profiled here may rely on this metabolic plasticity for growth and survival when certain resources are limiting.

The construction of these metagenomes also highlights how little is known about the effects of NO_3_- pollution on microbial communities, and the relationship between community stability and function in response to disturbance. Future research could begin to unravel the importance of stress tolerance and fermentation for microbial survival following short-term exposure to NO_3_-. In addition, future studies on the presence of *Acidobacteria*, a group that is understudied as a whole, in high NO_3_- conditions can also help to understand the distribution of this taxonomic group.

## Methods

### Sample preparation

Vernal pool microcosms were replicated in 500 mL glass jars by adding 50 g of soil collected from four vernal pools located in a temperate deciduous forest of Northeast Ohio, USA. The soil was air dried and sieved to remove extraneous matter and mixed with 50 g of autoclaved coarse sand to prevent excessive compaction of the soil media prior to addition to the microcosms. Each microcosm received 800 mg of dried leaf discs on the surface of the soil media and 150 mL of sterile water. Throughout the experiment, the microcosms were held in an incubator with a 12/12 hour day night cycle, with temperatures between 15–17°C to mimic spring forest conditions. The microcosms were subjected to an initial pH manipulation (5, 6, 7, or 8) on day zero and N addition on day 30 (D30). This experimental design was used to simulate persistent pH changes previously observed in vernal pools across an urbanization gradient [7] and NO_3_- pulses that are often associated with polluted runoff [48], which can be a significant source of input into vernal pools. While the complete experiment contained 72 microcosms and full details of the experimental set up are described elsewhere [17], a subset of 12 microcosms were used for the metagenomic analysis reported here and were those that were manipulated to a pH of 6.0 ± 0.3 at the beginning of the experiment and received either an addition of 10 mg NO_3_-_-_N or an equal volume of distilled water as a control on D30. There were six replicate microcosms for each treatment (NO_3_- addition and control). The NO_3_- addition and distilled water treatments were used because denitrification rate differed in these microcosms (an average of 3.84 ± 0.44 mg N (kg soil)^-1^ day^-1^ when NO_3_- was added and not detected in the microcosms receiving distilled water) [17]. Two replicate soil samples were collected and pooled from each microcosm on D30 approximately 20 hours after the NO_3_- addition and frozen at −70°C until used for DNA extraction. Soil samples were further pooled by combining 125 mg of soil from two replicate microcosms in the same treatment and then subjecting this pooled soil sample to DNA extraction as described elsewhere [17]. Therefore, there were three replicate DNA samples for each treatment that were used to create two metagenomes: one for the nitrate treatment (labeled +NO_3_-) and one for the distilled water treatment (labeled --N).

### Pyrosequencing

Similar to other shotgun metagenomic studies [20,49-51], DNA was amplified with the illustra Genomiphi V2 amplification kit (GE Healthcare Life Sciences, Inc., Piscataway, NJ) following the manufacturer’s protocol. Two replicate Genomiphi reactions were prepared for each microcosm DNA sample, making six reactions total for each treatment (three replicate microcosm DNA samples × two replicate Genomiphi reactions). The Genomiphi reactions randomly amplified regions of genomic DNA using primers of random sequences and resulted in 8 μg of amplified DNA from the +NO_3_- sample and the 10 μg of amplified DNA from the –N sample. Because of the use of random primers, these amplified DNA samples potentially included segments of DNA from all microbial species present in the samples and from regions throughout the microbial genomes. The amplified DNA from Genomiphi reactions was precipitated with sodium acetate and purified with 80% cold ethanol before being sent to Inqaba Biotec (Pretoria, South Africa) for 454 pyrosequencing on a GS-FLX platform.

### Sequence analysis

Because the metagenomes constructed from our microcosms contained DNA reads from multiple species, they were analyzed unassembled using the MG-RAST server [18] and are publicly available with the MG-RAST ID numbers 4445106.3 (+NO_3_-) and 4445130.3 (−N). Metagenomes are also available through the NCBI site [GenBank: SRP005560]. A BLASTX comparison to a non-redundant protein database was used to match the EGTs in the metagenomes to SEED subsystems [19]. The SEED protein-coding database has been used successfully for comparing shotgun metagenomes to taxonomic [20,21,51] and metabolic sequences [20,21,49-51] in environmental samples. Only matches that had an e-value of 10^-5^ or lower and had sequence similarity of 50 base pairs or greater were included in our MG-RAST analysis.

Metagenomes were also analyzed with a local BLASTN to a database of N metabolism genes that we constructed with searches at the NCBI site. The database included the known genes for the enzymes involved in denitrification, DNRA, and Annamox (using [12,52] as guides for the genes to include), as these processes are nitrate reduction pathways. The highly profiled functional genes for nitrification (*amoA*, *amoB*, and *amoC*) and nitrogen fixation (*nifD*, *nifH*, and *nifK*) were also included. The database contained a total of 111,502 sequences and a complete list of the genes included in the database can be found in Additional file 2: Table S5. The searches for the genes to include in the database at the NCBI site were to the “Nucleotide” collection of the International Nucleotide Sequence Database Collaboration (DDBJ/EMBL/GenBank) with limits, which excluded sequence tagged sites (STSs), third party annotation (TPA) sequences, high throughput genomic (HTG) sequences, patents, and whole genome shotgun (WGS) sequences. Additional limits were that the search field was gene name and the molecule was genomic DNA/RNA., We also excluded hits that included “complete genome” in any field. (The search field was as follows: “xxxX [Gene Name] AND biol_genomic [PROP] NOT “complete genome” [All Fields]”, where “xxxX” corresponds to the gene that was being searched for, such as “*nosZ*”.) The local BLASTN was conducted at Case Western Reserve University’s Genome and Transcriptome Analysis Core facility. A number of sequences in our database were complete chromosome sequences that included genes other than the N metabolism genes we were interested in. If sequences from the metagenomes matched with these database entries, they were only retained if the gene region of the BLASTN match was to a N metabolism gene of interest (e.g., if the match between the metagenome sequence and the database entry was to the gene region coding for a N metabolism gene of interest, such as the *napA* gene, it was kept, but if the match was to a non-N metabolism gene, such as the *trpS* gene, it was removed.) The BLASTN comparison included an e-value cutoff of 10^-5^ or lower and sequence similarity cutoff of 50 base pairs or greater.

### Statistical analysis

The Statistical Analysis of Metagenomic Profiles (STAMP) program was used to compare the +NO_3_- and –N metagenomes by identifying the proportional representation of different metabolic or phylogenetic groups and determining if they were statistically different between the two metagenomes with two-sided Fisher exact tests [53]. The MG-RAST functional matches at all levels and taxonomic matches at the class level and higher were compared with Fisher exact tests. Storey’s false discovery rate (FDR) method was applied to the Fisher exact tests as a multiple comparison test correction, resulting in q-values, which are the FDR equivalent of p-values. Confidence intervals were determined with the Newcome-Wilson method at α = 0.05. Statistically significant features that had less than five sequences or low effect sizes (<0.5 difference between proportions or <1.0 ratio of proportions) were removed from the analysis. In addition, a two sided chi-square test, with Yates’ correction for continuity, was conducted, also using STAMP, on the level two subsystems. This test was done specifically to investigate if any level two EGTs in the N metabolism category were statistically different with a less conservative test [53]. Confidence intervals were calculated and effect size filters were used as with the Fisher exact tests. The multiple comparison test correction used was the Benjamini-Hochberg FDR. Only biologically meaningful categories were included in the results reported here (i.e., the miscellaneous category for subsystems was removed and, for the phylogenetic EGT matches, unclassified taxonomic groups were removed).

## Competing interests

The authors declare that they have no competing interests.

## Authors’ contributions

SC-K conceived of the study, collected and processed samples for sequencing, and authored the manuscript. KS participated in the design and implementation of the study and edited and commented on the paper. DB conceived of the study and participated in its design and implementation, contributed to data analysis, and edited and commented on the paper. All authors read and approved the final manuscript.

## Supplementary Material

Additional file 1: Tables S1-S4 Results from Fisher exact tests at all subsystem levels and a chi-square test conducted at level two using the Statistical Analysis of Metagenomic Profiles program. Click here for file

Additional file 2: Tables S5-S6 Nitrogen metabolism genes included in the database created from the NCBI site and all matches from the +NO_3_- metagenome to nitrogen metabolism genes with a BLASTN. Click here for file
