# Supplementary material for: Shotgun metagenomic analysis of metabolic diversity and microbial community structure in experimental vernal pools subjected to nitrate pulse
Source: BMC Microbiol. 2013 Apr 10;13:78. doi: 10.1186/1471-2180-13-78 (PMC3629998; doi:10.1186/1471-2180-13-78)
Supplement: Additional file 1: Tables S1-S4 — Results from Fisher exact tests at all subsystem levels and a chi-square test conducted at level two using the Statistical Analysis of Metagenomic Profiles program. [file 1471-2180-13-78-S1.doc]

**Table S1: Results of a Fisher exact test at the lowest subsystem level of function. Only environmental gene tag (EGT) matches that show significant differences between the +NO3- and –N metagenomes are reported here.**

|  | **EGT Match** | | | **Proportional Representation (%)** | | **Corrected p-value** | **Effect size** |
| --- | --- | --- | --- | --- | --- | --- | --- |
| **Subsystem Category** | **Level 2** | **Level 3** | **Function** | **+NO_3_^-^** | **--N** |
| Amino Acids and Derivatives | Alanine, serine, and glycine | Alanine biosynthesis | Cysteine desulfurase (EC 2.8.1.7), SufS subfamily | 0.65 | 0 | 2.51E-20 | 0.65 |
| Amino Acids and Derivatives | Alanine, serine, and glycine | Alanine biosynthesis | Cysteine desulfurase (EC 2.8.1.7) | 1.62 | 2.39 | 0.0024 | -0.77 |
| Carbohydrates | CO2 fixation | Calvin-Benson cycle | NAD-dependent glyceraldehyde-3-phosphate dehydrogenase (EC 1.2.1.12) | 1.55 | 0.25 | 3.55E-23 | 1.30 |
| Clustering-based subsystems | CBSS-296591.1.peg.2330 | - | UDP-glucose 4-epimerase (EC 5.1.3.2) | 0 | 0.85 | 4.96E-20 | -0.85 |
| Clustering-based subsystems | CBSS-235.1.peg.567 | - | 2-amino-4-hydroxy-6-hydroxymethyldihydropteridine pyrophosphokinase (EC 2.7.6.3) | 0.54 | 0 | 4.85E-17 | 0.54 |
| Fatty Acids, Lipids, and Isoprenoids | Phospholipids | Glycerolipid and Glycerophospholipid Metabolism in Bacteria | Aldehyde dehydrogenase (EC 1.2.1.3) | 0.85 | 0 | 8.17E-27 | 0.85 |
| Iron acquisition and metabolism | Iron acquisition in Vibrio | - | TonB-dependent receptor | 0 | 0.75 | 1.03E-17 | -0.75 |
| Protein Metabolism | Protein processing and modification | G3E family of P-loop GTPases (metallocenter biosynthesis) | Urease beta subunit (EC 3.5.1.5) | 0 | 0.82 | 1.56E-19 | -0.82 |
| Protein Metabolism | Protein processing and modification | G3E family of P-loop GTPases (metallocenter biosynthesis) | Urease alpha subunit (EC 3.5.1.5) | 0.11 | 0.91 | 2.99E-13 | -0.80 |
| Stress Response | Oxidative stress | Oxidative stress | Alkyl hydroperoxide reductase subunit C-like protein | 1.22 | 0.17 | 6.16E-20 | 1.04 |

**Table S2: Results of a Fisher exact test at the subsystem level 3. Only environmental gene tag (EGT) matches that show significant differences between the +NO3- and –N metagenomes are reported here.**

|  | **EGT Match** | | **Proportional Representation (%)** | |  |  |
| --- | --- | --- | --- | --- | --- | --- |
| **Subsystem Category** | **Level 2** | **Level 3** | **+NO_3_^-^** | **--N** | **Corrected p-value** | **Effect size** |
| Carbohydrates | CO2 fixation | Calvin-Benson cycle | 2.59 | 1.54 | 4.06E-06 | 1.049 |
| Carbohydrates | Fermentation | Acetyl-CoA fermentation to Butyrate | 1.88 | 1.24 | 0.0022 | 0.63 |
| Cofactors, Vitamins, Prosthetic Groups, Pigments | Folate and pterines | 5-FCL-like protein | 3.55 | 2.79 | 0.012 | 0.76 |
| Fatty Acids, Lipids, and Isoprenoids | Phospholipids | Glycerolipid and Glycerophospholipid Metabolism in Bacteria | 0.97 | 0.078 | 1.10E-20 | 0.89 |
| Protein Metabolism | Protein processing and modification | G3E family of P-loop GTPases (metallocenter biosynthesis) | 0.73 | 2.15 | 1.39E-14 | -1.43 |
| Stress Response | Oxidative stress | Oxidative stress | 1.71 | 0.58 | 8.07E-13 | 1.13 |
| Virulence, Disease and Defense | Resistance to antibiotics and toxic compounds | Cobalt-zinc-cadmium resistance | 1.93 | 1.31 | 0.0032 | 0.62 |

**Table S3: Results of a Fisher exact test at the subsystem level 2. Only environmental gene tag (EGT) matches that show significant differences between the +NO3- and –N metagenomes are reported here.**

|  |  | **Proportional Representation (%)** | |  |  |
| --- | --- | --- | --- | --- | --- |
| **Subsystem Category** | **Level 2 EGT Match** | **+NO_3_^-^** | **--N** | **Corrected p-value** | **Effect size** |
| Amino Acids and Derivatives | Aromatic amino acids and derivatives | 0.66 | 1.31 | 7.91E-05 | -0.65 |
| Amino Acids and Derivatives | Branched-chain amino acids | 2.27 | 1.73 | 0.028 | 0.54 |
| Carbohydrates | CO2 fixation | 2.89 | 1.60 | 2.07E-08 | 1.29 |
| Carbohydrates | Fermentation | 4.46 | 3.32 | 0.00033 | 1.14 |
| Cell Wall and Capsule | Gram-Negative cell wall components | 1.62 | 1.060 | 0.0035 | 0.56 |
| Clustering-based subsystems | CBSS-296591.1.peg.2330 | 0.65 | 1.53 | 1.57E-07 | -0.88 |
| Fatty Acids, Lipids, and Isoprenoids | Phospholipids | 0.97 | 0.078 | 1.42E-20 | 0.89 |
| Fatty Acids, Lipids, and Isoprenoids | Isoprenoids | 1.040 | 0.49 | 6.88E-05 | 0.55 |
| Iron acquisition and metabolism | Iron acquisition in Vibrio | 0.027 | 0.75 | 2.88E-15 | -0.73 |
| Protein Metabolism | Protein processing and modification | 0.88 | 2.23 | 3.82E-12 | -1.36 |
| Regulation and Cell signaling | cAMP signaling in bacteria | 0.69 | 0.18 | 3.81E-07 | 0.50 |
| Regulation and Cell signaling | Regulation of virulence | 0.35 | 0.95 | 5.68E-06 | -0.59 |
| RNA Metabolism | RNA processing and modification | 1.66 | 2.70 | 1.52E-05 | -1.034 |
| Stress Response | Oxidative stress | 1.81 | 0.86 | 1.01E-07 | 0.95 |

**Table S4: Results of a chi-square test at the subsystem level 2. Only environmental gene tag (EGT) matches that show significant differences between the +NO3- and –N metagenomes are reported here.**

|  |  | **Proportional Representation (%)** | |  |  |
| --- | --- | --- | --- | --- | --- |
| **Subsystem Category** | **Level 2 EGT Match** | **+NO_3_^-^** | **--N** | **Corrected p-value** | **Effect size** |
| Amino Acids and Derivatives | Aromatic amino acids and derivatives | 0.66 | 1.31 | 0.00029 | -0.65 |
| Amino Acids and Derivatives | Branched-chain amino acids | 2.27 | 1.73 | 0.044 | 0.54 |
| Carbohydrates | CO2 fixation | 2.89 | 1.60 | 2.22E-08 | 1.29 |
| Carbohydrates | Fermentation | 4.46 | 3.32 | 0.00047 | 1.14 |
| Cell Wall and Capsule | Gram-Negative cell wall components | 1.62 | 1.060 | 0.0055 | 0.56 |
| Clustering-based subsystems | CBSS-296591.1.peg.2330 | 0.65 | 1.53 | 1.15E-06 | -0.88 |
| Fatty Acids, Lipids, and Isoprenoids | Phospholipids | 0.97 | 0.078 | 0 | 0.89 |
| Fatty Acids, Lipids, and Isoprenoids | Isoprenoids | 1.040 | 0.49 | 8.04E-05 | 0.55 |
| Iron acquisition and metabolism | Iron acquisition in Vibrio | 0.027 | 0.75 | 1.69E-10 | -0.73 |
| Protein Metabolism | Protein processing and modification | 0.88 | 2.23 | 1.65E-10 | -1.36 |
| Regulation and Cell signaling | cAMP signaling in bacteria | 0.69 | 0.18 | 3.73E-07 | 0.50 |
| Regulation and Cell signaling | Regulation of virulence | 0.35 | 0.95 | 4.00E-05 | -0.59 |
| RNA Metabolism | RNA processing and modification | 1.66 | 2.70 | 4.31E-05 | -1.03 |
| Stress Response | Oxidative stress | 1.81 | 0.86 | 7.73E-08 | 0.95 |
