# Supplementary material for: Shotgun metagenomic analysis of metabolic diversity and microbial community structure in experimental vernal pools subjected to nitrate pulse
Source: BMC Microbiol. 2013 Apr 10;13:78. doi: 10.1186/1471-2180-13-78 (PMC3629998; doi:10.1186/1471-2180-13-78)
Supplement: Additional file 2: Tables S5-S6 — Nitrogen metabolism genes included in the database created from the NCBI site and all matches from the +NO3- metagenome to nitrogen metabolism genes with a BLASTN. [file 1471-2180-13-78-S2.doc]

**Table S5: Nitrogen metabolism genes included in the nitrogen metabolism database created from the NCBI site.**

| **N cycling pathway** | **Gene** | **Product** | **Number of sequences in the database** |
| --- | --- | --- | --- |
| Denitrification | *narG* | nitrate reductase | 2865 |
|  | *napA* | nitrate reductase | 720 |
|  | *nirS* | nitrite reductase | 9566 |
|  | *nirK* | nitrite reductase | 7881 |
|  | *norB* | nitric oxide reductase | 208 |
|  | *norZ* | nitric oxide reductase | 5 |
|  | *nosZ* | nitrous oxide reductase | 5917 |
| DNRA | *napA* | nitrate reductase | (done for denitrification) |
|  | *napB* | nitrate reductase | 56 |
|  | *napC* | nitrate reductase | 22 |
|  | *napD* | nitrate reductase | 24 |
|  | *napE* | nitrate reductase | 17 |
|  | *napF* | nitrate reductase | 18 |
|  | *napG* | nitrate reductase | 14 |
|  | *napH* | nitrate reductase | 10 |
|  | *napK* | nitrate reductase | 2 |
|  | *napL* | nitrate reductase | 1 |
|  | *nrfA* | nitrite reductase | 70 |
|  | *nrfB* | nitrite reductase | 5 |
|  | *nrfC* | nitrite reductase | 24 |
|  | *nrfD* | nitrite reductase | 7 |
|  | *nrfH* | nitrite reductase | 5 |
|  | *nrfI* | nitrite reductase | 2 |
|  | *nrfJ* | nitrite reductase | 1 |
| Annamox | *narG* | nitrate reductase | (done for denitrification) |
|  | *narH* | nitrate reductase | 89 |
|  | *nirS* | nitrite reductase | (done for denitrification) |
|  | *hao/hzo* | anammox-specific hydroxylamine oxidoreductase | 15 |
|  | *hzo* | hydrazine-oxidizing enzyme | 1404 |
| N fixation | *nifH* | nitrogenase | 31,718 |
|  | *nifK* | nitrogenase | 334 |
|  | *nifD* | nitrogenase | 1202 |
| Nitrification | *amoA* | ammonia monooxygenase | 48,767 |
|  | *amoB* | ammonia monooxygenase | 97 |
|  | *amoC* | ammonia monooxygenase | 30 |
|  | *hao* | hydroxylamine oxidoreductase | 179 |
|  | *nxrA* | nitrite oxidoreductase | 227 |
|  |  |  | **Total sequence number in database = 111,502**^1^ |

^1^Some database entries were the same sequence from the NCBI site, as they contained multiple nitrogen metabolism genes that were searched for.

**Table S6: Nitrogen metabolism gene matches with the +NO_3_^-^ metagenome.**

| **Query Sequence**^1^ | **Database Match** | | **%ID** | **Align Length** | **E-value** | **Gene match**^2^  **(region)** |
| --- | --- | --- | --- | --- | --- | --- |
|  | **Accession #** | **Description** |  |  |  |  |
| +NO_3_^-^ seq. 1 | FP885891.2 | Ralstonia solanacearum PSI07 megaplasmid mpPSI07, complete sequence | 92.42 | 66 | 1.00E-17 | *napA*  (1522911-1525652) |
| +NO_3_^-^ seq. 1 | FR854064.1 | blood disease bacterium R229, genomic contig 00008-1626 | 92.42 | 66 | 1.00E-17 | *napA*  (257824-260565) |
| +NO_3_^-^ seq. 1 | FR854092.1 | Ralstonia syzygii R24, genomic contig 00003-1629 | 93.65 | 63 | 2.00E-18 | *napA*  (153253-155994) |
| +NO_3_^-^ seq. 2 | EF217059.1 | Uncultured bacterium clone CSA1 putitative periplasmic nitrate reductase large subunit (napA) gene, partial cds | 88.14 | 59 | 1.00E-08 |  |
| +NO_3_^-^ seq. 2 | EF217062.1 | Uncultured bacterium clone CSA4 putitative periplasmic nitrate reductase large subunit (napA) gene, partial cds | 82.89 | 152 | 7.00E-19 |  |
| +NO_3_^-^ seq. 2 | EF217063.1 | Uncultured bacterium clone CSA5 putitative periplasmic nitrate reductase large subunit (napA) gene, partial cds | 80.92 | 152 | 1.00E-11 |  |
| +NO_3_^-^ seq. 2 | EF217064.1 | Uncultured bacterium clone CSA6 putitative periplasmic nitrate reductase large subunit (napA) gene, partial cds | 81.82 | 143 | 2.00E-13 |  |
| +NO_3_^-^ seq. 2 | EF217065.1 | Uncultured bacterium clone CSA7 putitative periplasmic nitrate reductase large subunit (napA) gene, partial cds | 81.43 | 140 | 1.00E-11 |  |
| +NO_3_^-^ seq. 2 | EF217066.1 | Uncultured bacterium clone CSA8 putitative periplasmic nitrate reductase large subunit (napA) gene, partial cds | 81.88 | 149 | 1.00E-14 |  |
| +NO_3_^-^ seq. 2 | EF217067.1 | Uncultured bacterium clone CSA9 putitative periplasmic nitrate reductase large subunit (napA) gene, partial cds | 82.55 | 149 | 5.00E-17 |  |
| +NO_3_^-^ seq. 2 | EF217068.1 | Uncultured bacterium clone CSA10 putitative periplasmic nitrate reductase large subunit (napA) gene, partial cds | 81.82 | 143 | 2.00E-13 |  |
| +NO_3_^-^ seq. 2 | EF217069.1 | Uncultured bacterium clone CSA11 putitative periplasmic nitrate reductase large subunit (napA) gene, partial cds | 81.82 | 143 | 2.00E-13 |  |
| +NO_3_^-^ seq. 2 | EF217070.1 | Uncultured bacterium clone CSA12 putitative periplasmic nitrate reductase large subunit (napA) gene, partial cds | 83.22 | 149 | 2.00E-19 |  |
| +NO_3_^-^ seq. 2 | EF217074.1 | Uncultured bacterium clone CSA16 putitative periplasmic nitrate reductase large subunit (napA) gene, partial cds | 80.92 | 152 | 1.00E-11 |  |
| +NO_3_^-^ seq. 2 | EF217075.1 | Uncultured bacterium clone CSA17 putitative periplasmic nitrate reductase large subunit (napA) gene, partial cds | 81.58 | 152 | 4.00E-14 |  |
| +NO_3_^-^ seq. 2 | EF217076.1 | Uncultured bacterium clone CSA18 putitative periplasmic nitrate reductase large subunit (napA) gene, partial cds | 84.77 | 151 | 2.00E-25 |  |
| +NO_3_^-^ seq. 2 | EF217077.1 | Uncultured bacterium clone CSA19 putitative periplasmic nitrate reductase large subunit (napA) gene, partial cds | 81.88 | 149 | 1.00E-14 |  |
| +NO_3_^-^ seq. 2 | EF217078.1 | Uncultured bacterium clone CSA20 putitative periplasmic nitrate reductase large subunit (napA) gene, partial cds | 82.52 | 143 | 7.00E-16 |  |
| +NO_3_^-^ seq. 2 | EF217079.1 | Uncultured bacterium clone CSA21 putitative periplasmic nitrate reductase large subunit (napA) gene, partial cds | 82.14 | 140 | 4.00E-14 |  |
| +NO_3_^-^ seq. 2 | EF217082.1 | Uncultured bacterium clone CSA24 putitative periplasmic nitrate reductase large subunit (napA) gene, partial cds | 85.14 | 74 | 4.00E-08 |  |
| +NO_3_^-^ seq. 2 | EF217083.1 | Uncultured bacterium clone CSA25 putitative periplasmic nitrate reductase large subunit (napA) gene, partial cds | 88.68 | 53 | 2.00E-07 |  |
| +NO_3_^-^ seq. 2 | EF217084.1 | Uncultured bacterium clone CSA26 putitative periplasmic nitrate reductase large subunit (napA) gene, partial cds | 81.88 | 149 | 1.00E-14 |  |
| +NO_3_^-^ seq. 2 | EF217085.1 | Uncultured bacterium clone CSA27 putitative periplasmic nitrate reductase large subunit (napA) gene, partial cds | 81.88 | 149 | 1.00E-14 |  |
| +NO_3_^-^ seq. 2 | EF217089.1 | Uncultured bacterium clone CSA31 putitative periplasmic nitrate reductase large subunit (napA) gene, partial cds | 82.61 | 92 | 6.00E-07 |  |
| +NO_3_^-^ seq. 2 | EF217090.1 | Uncultured bacterium clone Gar1 putitative periplasmic nitrate reductase large subunit (napA) gene, partial cds | 81.21 | 149 | 3.00E-12 |  |
| +NO_3_^-^ seq. 2 | EF217091.1 | Uncultured bacterium clone Gar2 putitative periplasmic nitrate reductase large subunit (napA) gene, partial cds | 81.21 | 149 | 3.00E-12 |  |
| +NO_3_^-^ seq. 2 | EF217093.1 | Uncultured bacterium clone Gar4 putitative periplasmic nitrate reductase large subunit (napA) gene, partial cds | 82.55 | 149 | 5.00E-17 |  |
| +NO_3_^-^ seq. 2 | EF217102.1 | Uncultured bacterium clone YVET2 putitative periplasmic nitrate reductase large subunit (napA) gene, partial cds | 80.92 | 152 | 1.00E-11 |  |
| +NO_3_^-^ seq. 2 | EF217105.1 | Uncultured bacterium clone YVET5 putitative periplasmic nitrate reductase large subunit (napA) gene, partial cds | 83.89 | 149 | 8.00E-22 |  |
| +NO_3_^-^ seq. 2 | EF217107.1 | Uncultured bacterium clone PAD1 putitative periplasmic nitrate reductase large subunit (napA) gene, partial cds | 90.32 | 62 | 7.00E-13 |  |
| +NO_3_^-^ seq. 2 | EF217108.1 | Uncultured bacterium clone PAD2 putitative periplasmic nitrate reductase large subunit (napA) gene, partial cds | 88.71 | 62 | 2.00E-10 |  |
| +NO_3_^-^ seq. 2 | EF217110.1 | Uncultured bacterium clone PAD4 putitative periplasmic nitrate reductase large subunit (napA) gene, partial cds | 87.1 | 62 | 4.00E-08 |  |
| +NO_3_^-^ seq. 2 | EF217113.1 | Uncultured bacterium clone PAD7 putitative periplasmic nitrate reductase large subunit (napA) gene, partial cds | 91.38 | 58 | 7.00E-13 |  |
| +NO_3_^-^ seq. 2 | |EF217119.1 | Uncultured bacterium clone PAD13 putitative periplasmic nitrate reductase large subunit (napA) gene, partial cds | 86.15 | 65 | 2.00E-07 |  |
| +NO_3_^-^ seq. 2 | EF217122.1 | Uncultured bacterium clone Rotm1 putitative periplasmic nitrate reductase large subunit (napA) gene, partial cds | 80.92 | 152 | 1.00E-11 |  |
| +NO_3_^-^ seq. 2 | EF217123.1 | Uncultured bacterium clone Rotm2 putitative periplasmic nitrate reductase large subunit (napA) gene, partial cds | 83.13 | 83 | 3.00E-06 |  |
| +NO_3_^-^ seq. 2 | EF217130.1 | Uncultured bacterium clone Rotm10 putitative periplasmic nitrate reductase large subunit (napA) gene, partial cds | 85.14 | 74 | 4.00E-08 |  |
| +NO_3_^-^ seq. 2 | EF217131.1 | Uncultured bacterium clone Rotm11 putitative periplasmic nitrate reductase large subunit (napA) gene, partial cds | 82.55 | 149 | 5.00E-17 |  |
| +NO_3_^-^ seq. 2 | EF217133.1 | Uncultured bacterium clone Rotm13 putitative periplasmic nitrate reductase large subunit (napA) gene, partial cds | 81.82 | 99 | 3.00E-06 |  |
| +NO_3_^-^ seq. 2 | EF217134.1 | Uncultured bacterium clone Rotm14 putitative periplasmic nitrate reductase large subunit (napA) gene, partial cds | 81.82 | 99 | 3.00E-06 |  |
| +NO_3_^-^ seq. 2 | AB055444.2 | Magnetospirillum magnetotacticum periplasmic nitrate reductase gene cluster (napF, napD, napA, napG, napH, napB, napC), complete cds | 88.16 | 76 | 4.00E-14 | *napA*  (1335-3818) |
| +NO_3_^-^ seq. 2 | AB196638.1 | Pseudomonas sp. MT-1 orf5, orf6, napD, napA, napB, napC, orf7 genes, partial and complete cds | 79.6 | 201 | 3.00E-12 | *napA*  (2333-4837) |
| +NO_3_^-^ seq. 2 | EF645075.1 | Uncultured bacterium clone A30P54 putative periplasmic nitrate reductase (napA) gene, partial cds | 85.94 | 64 | 6.00E-07 |  |
| +NO_3_^-^ seq. 2 | EF645108.1 | Uncultured bacterium clone A30P62 putative periplasmic nitrate reductase (napA) gene, partial cds | 90.77 | 65 | 1.00E-14 |  |
| +NO_3_^-^ seq. 2 | EF645116.1 | Uncultured bacterium clone A30P43 putative periplasmic nitrate reductase (napA) gene, partial cds | 89.06 | 64 | 1.00E-11 |  |
| +NO_3_^-^ seq. 2 | EF645118.1 | Uncultured bacterium clone A30J15 putative periplasmic nitrate reductase (napA) gene, partial cds | 89.23 | 65 | 3.00E-12 |  |
| +NO_3_^-^ seq. 2 | EF645131.1 | Uncultured bacterium clone A30K24 putative periplasmic nitrate reductase (napA) gene, partial cds | 89.83 | 59 | 4.00E-11 |  |
| +NO_3_^-^ seq. 2 | EU495707.1 | Uncultured bacterium clone T3-B02 catalytic subunit of periplasmic nitrate reductase (napA) gene, partial cds | 78.68 | 197 | 2.00E-07 |  |
| +NO_3_^-^ seq. 2 | EU495721.1 | Uncultured bacterium clone T3-C06 catalytic subunit of periplasmic nitrate reductase (napA) gene, partial cds | 78.68 | 197 | 2.00E-07 |  |
| +NO_3_^-^ seq. 2 | EU495726.1 | Uncultured bacterium clone T3-C12 catalytic subunit of periplasmic nitrate reductase (napA) gene, partial cds | 78.68 | 197 | 2.00E-07 |  |
| +NO_3_^-^ seq. 2 | EU495728.1 | Uncultured bacterium clone T3-D04 catalytic subunit of periplasmic nitrate reductase (napA) gene, partial cds | 78.68 | 197 | 2.00E-07 |  |
| +NO_3_^-^ seq. 2 | EU495739.1 | Uncultured bacterium clone T3-E05 catalytic subunit of periplasmic nitrate reductase (napA) gene, partial cds | 79.19 | 197 | 7.00E-10 |  |
| +NO_3_^-^ seq. 2 | EU495751.1 | Uncultured bacterium clone T3-F08 catalytic subunit of periplasmic nitrate reductase (napA) gene, partial cds | 78.68 | 197 | 2.00E-07 |  |
| +NO_3_^-^ seq. 2 | EU495752.1 | Uncultured bacterium clone T3-F09 catalytic subunit of periplasmic nitrate reductase (napA) gene, partial cds | 78.68 | 197 | 2.00E-07 |  |
| +NO_3_^-^ seq. 2 | EU495769.1 | Uncultured bacterium clone T3-H09 catalytic subunit of periplasmic nitrate reductase (napA) gene, partial cds | 78.68 | 197 | 2.00E-07 |  |
| +NO_3_^-^ seq. 2 | EU495650.1 | Uncultured bacterium clone T1-E01 NapA (napA) gene, partial cds | 82.91 | 199 | 8.00E-28 |  |
| +NO_3_^-^ seq. 2 | EU495652.1 | Uncultured bacterium clone T1-E04 NapA (napA) gene, partial cds | 82.05 | 195 | 5.00E-23 |  |
| +NO_3_^-^ seq. 2 | EU495653.1 | Uncultured bacterium clone T1-E05 NapA (napA) gene, partial cds | 85.86 | 99 | 7.00E-16 |  |
| +NO_3_^-^ seq. 2 | EU495656.1 | Uncultured bacterium clone T1-E08 NapA (napA) gene, partial cds | 82.05 | 195 | 5.00E-23 |  |
| +NO_3_^-^ seq. 2 | EU495657.1 | Uncultured bacterium clone T1-E09 NapA (napA) gene, partial cds | 80.4 | 199 | 7.00E-16 |  |
| +NO_3_^-^ seq. 2 | EU495659.1 | Uncultured bacterium clone T1-E11 NapA (napA) gene, partial cds | 81.98 | 111 | 1.00E-08 |  |
| +NO_3_^-^ seq. 2 | EU495660.1 | Uncultured bacterium clone T1-E12 NapA (napA) gene, partial cds | 80.83 | 193 | 5.00E-17 |  |
| +NO_3_^-^ seq. 2 | EU495661.1 | Uncultured bacterium clone T1-F01 NapA (napA) gene, partial cds | 84.07 | 113 | 1.00E-14 |  |
| +NO_3_^-^ seq. 2 | EU495662.1 | Uncultured bacterium clone T1-F02 NapA (napA) gene, partial cds | 82.35 | 102 | 4.00E-08 |  |
| +NO_3_^-^ seq. 2 | EU495663.1 | Uncultured bacterium clone T1-F03 NapA (napA) gene, partial cds | 78.97 | 195 | 1.00E-08 |  |
| +NO_3_^-^ seq. 2 | EU495665.1 | Uncultured bacterium clone T1-F05 NapA (napA) gene, partial cds | 81.12 | 143 | 4.00E-11 |  |
| +NO_3_^-^ seq. 2 | EU495666.1 | Uncultured bacterium clone T1-F06 NapA (napA) gene, partial cds | 82.56 | 172 | 3.00E-21 |  |
| +NO_3_^-^ seq. 2 | EU495668.1 | Uncultured bacterium clone T1-F08 NapA (napA) gene, partial cds | 81.98 | 172 | 7.00E-19 |  |
| +NO_3_^-^ seq. 2 | EU495669.1 | Uncultured bacterium clone T1-F09 NapA (napA) gene, partial cds | 81.59 | 201 | 8.00E-22 |  |
| +NO_3_^-^ seq. 2 | EU495670.1 | Uncultured bacterium clone T1-F10 NapA (napA) gene, partial cds | 81.86 | 215 | 2.00E-25 |  |
| +NO_3_^-^ seq. 2 | EU495671.1 | Uncultured bacterium clone T1-F11 NapA (napA) gene, partial cds | 79.66 | 177 | 7.00E-10 |  |
| +NO_3_^-^ seq. 2 | EU495672.1 | Uncultured bacterium clone T1-F12 NapA (napA) gene, partial cds | 82.41 | 199 | 2.00E-25 |  |
| +NO_3_^-^ seq. 2 | EU495673.1 | Uncultured bacterium clone T1-G01 NapA (napA) gene, partial cds | 81.98 | 111 | 1.00E-08 |  |
| +NO_3_^-^ seq. 2 | EU495674.1 | Uncultured bacterium clone T1-G02 NapA (napA) gene, partial cds | 85.86 | 99 | 7.00E-16 |  |
| +NO_3_^-^ seq. 2 | EU495675.1 | Uncultured bacterium clone T1-G03 NapA (napA) gene, partial cds | 84.44 | 90 | 2.00E-10 |  |
| +NO_3_^-^ seq. 2 | EU495677.1 | Uncultured bacterium clone T1-G05 NapA (napA) gene, partial cds | 86.46 | 96 | 2.00E-16 |  |
| +NO_3_^-^ seq. 2 | EU495678.1 | Uncultured bacterium clone T1-G06 NapA (napA) gene, partial cds | 79.87 | 154 | 4.00E-08 |  |
| +NO_3_^-^ seq. 2 | EU495680.1 | Uncultured bacterium clone T1-G08 NapA (napA) gene, partial cds | 82.41 | 216 | 2.00E-28 |  |
| +NO_3_^-^ seq. 2 | EU495681.1 | Uncultured bacterium clone T1-G09 NapA (napA) gene, partial cds | 80 | 185 | 3.00E-12 |  |
| +NO_3_^-^ seq. 2 | EU495682.1 | Uncultured bacterium clone T1-G10 NapA (napA) gene, partial cds | 82.5 | 200 | 5.00E-26 |  |
| +NO_3_^-^ seq. 2 | EU495685.1 | Uncultured bacterium clone T1-H02 NapA (napA) gene, partial cds | 80.52 | 154 | 2.00E-10 |  |
| +NO_3_^-^ seq. 2 | EU495686.1 | Uncultured bacterium clone T1-H03 NapA (napA) gene, partial cds | 84.07 | 113 | 1.00E-14 |  |
| +NO_3_^-^ seq. 2 | EU495687.1 | Uncultured bacterium clone T1-H04 NapA (napA) gene, partial cds | 80.39 | 153 | 7.00E-10 |  |
| +NO_3_^-^ seq. 2 | EU495688.1 | Uncultured bacterium clone T1-H05 NapA (napA) gene, partial cds | 82.91 | 199 | 8.00E-28 |  |
| +NO_3_^-^ seq. 2 | EU495689.1 | Uncultured bacterium clone T1-H06 NapA (napA) gene, partial cds | 79.21 | 178 | 4.00E-08 |  |
| +NO_3_^-^ seq. 2 | EU495690.1 | Uncultured bacterium clone T1-H07 NapA (napA) gene, partial cds | 81.97 | 183 | 1.00E-20 |  |
| +NO_3_^-^ seq. 2 | EU495692.1 | Uncultured bacterium clone T1-H10 NapA (napA) gene, partial cds | 82.24 | 214 | 3.00E-27 |  |
| +NO_3_^-^ seq. 2 | AF245096.1 | Azospirillum brasilense periplasmic nitrate reductase large subunit (napA) gene, partial cds; and periplasmic nitrate reductase small diheme cytochrome c subunit (napB) and membrane-anchored tetraheme c-type cytochrome (napC) genes, complete cds | 94.55 | 55 | 7.00E-16 | *napA*  (1-2154) |
| +NO_3_^-^ seq. 2 | AF245096.1 | Azospirillum brasilense periplasmic nitrate reductase large subunit (napA) gene, partial cds; and periplasmic nitrate reductase small diheme cytochrome c subunit (napB) and membrane-anchored tetraheme c-type cytochrome (napC) genes, complete cds | 88.89 | 63 | 4.00E-11 | *napA*  (1-2154) |
| +NO_3_^-^ seq. 2 | AF314590.3 | Bradyrhizobium japonicum putative cyclic NTP-binding protein gene, partial cds; LuxA-like protein, MDO-like protein, and AraC-like protein genes, complete cds; nap operon, complete sequence; exodeoxyribosylnuclease EdrN (edrN) genes, complete cds; and putative sensor protein gene, partial cds | 82.61 | 253 | 4.00E-36 | *napA*  (5402-7918) |
| +NO_3_^-^ seq. 2 | AE006469.1 | Sinorhizobium meliloti 1021 plasmid pSymA, complete sequence | 84.03 | 119 | 7.00E-16 | *napA*  (677198-679702) |
| +NO_3_^-^ seq. 2 | AF069545.1 | Rhodobacter sphaeroides f. sp. denitrificans NapE (napE), NapF (napF), NapD (napD), periplasmic nitrate reductase precursor (napA), cytochrome c precursor (napB), and membranous tetrahemic cytochrome c (napC) genes, complete cds | 84.17 | 120 | 2.00E-16 | *napA*  (1168-3663) |
| +NO_3_^-^ seq. 2 | AY305378.1 | Ralstonia eutropha H16 megaplasmid pHG1, complete sequence | 82.86 | 105 | 7.00E-10 | *napA*  (228154-230649) |
| +NO_3_^-^ seq. 2 | AB016290.1 | Rhodobacter sphaeroides f. sp. denitrificans napK, napE, napF, napD, napA, napB and napC genes, partial and complete cds | 84.17 | 120 | 2.00E-16 | *napA*  (2067-4562) |
| +NO_3_^-^ seq. 2 | HQ727697.1 | Uncultured Pseudomonas sp. clone B-NO3-2 periplasmic nitrate reductase subunit (napA) gene, partial cds | 86.49 | 111 | 1.00E-20 |  |
| +NO_3_^-^ seq. 2 | HQ727698.1 | Uncultured Pseudomonas sp. clone B-NO3-1 periplasmic nitrate reductase subunit (napA) gene, partial cds | 86.49 | 111 | 1.00E-20 |  |
| +NO_3_^-^ seq. 2 | HQ727699. | Uncultured Pseudomonas sp. clone B-EVN-1 periplasmic nitrate reductase subunit (napA) gene, partial cds | 86.49 | 111 | 1.00E-20 |  |
| +NO_3_^-^ seq. 2 | HQ727701.1 | Uncultured Pseudomonas sp. clone B-ALK-1 periplasmic nitrate reductase subunit (napA) gene, partial cds | 85.39 | 89 | 3.00E-12 |  |
| +NO_3_^-^ seq. 2 | HQ727704.1 | Uncultured Thauera sp. clone M-NO3-6 periplasmic nitrate reductase subunit (napA) gene, partial cds | 85 | 120 | 7.00E-19 |  |
| +NO_3_^-^ seq. 2 | HQ727705.1 | Uncultured Thauera sp. clone M-NO3-7 periplasmic nitrate reductase subunit (napA) gene, partial cds | 85 | 120 | 7.00E-19 |  |
| +NO_3_^-^ seq. 2 | HQ727706.1 | Uncultured Thauera sp. clone M-NO3-5 periplasmic nitrate reductase subunit (napA) gene, partial cds | 85 | 120 | 7.00E-19 |  |
| +NO_3_^-^ seq. 2 | HQ727711.1 | Uncultured bacterium clone M-EVN-1 periplasmic nitrate reductase subunit (napA) gene, partial cds | 84.33 | 134 | 5.00E-20 |  |
| +NO_3_^-^ seq. 2 | HQ727712.1 | Uncultured Pseudomonas sp. clone M-ALK-1 periplasmic nitrate reductase subunit (napA) gene, partial cds | 88.29 | 111 | 2.00E-25 |  |
| +NO_3_^-^ seq. 2 | HQ727714.1 | Uncultured Thauera sp. clone M-ALK-2 periplasmic nitrate reductase subunit (napA) gene, partial cds | 85 | 120 | 7.00E-19 |  |
| +NO_3_^-^ seq. 2 | HQ727715.1 | Uncultured bacterium clone Z-ALK-2 periplasmic nitrate reductase subunit (napA) gene, partial cds | 86.11 | 144 | 2.00E-28 |  |
| +NO_3_^-^ seq. 2 | HQ727716.1 | Uncultured Cupriavidus sp. clone Z-NO3-2 periplasmic nitrate reductase subunit (napA) gene, partial cds | 82.86 | 105 | 7.00E-10 |  |
| +NO_3_^-^ seq. 2 | HQ727717.1 | Uncultured Thauera sp. clone Z-ALK-1 periplasmic nitrate reductase subunit (napA) gene, partial cds | 85.83 | 120 | 3.00E-21 |  |
| +NO_3_^-^ seq. 2 | HQ727718.1 | Uncultured Thauera sp. clone Z-NO3-1 periplasmic nitrate reductase subunit (napA) gene, partial cds | 85 | 120 | 7.00E-19 |  |
| +NO_3_^-^ seq. 2 | HQ727719.1 | Uncultured Pseudomonas sp. clone Z-EVN-2 periplasmic nitrate reductase subunit (napA) gene, partial cds | 85.39 | 89 | 3.00E-12 |  |
| +NO_3_^-^ seq. 2 | HQ727720.1 | Uncultured Thauera sp. clone Z-EVN-1 periplasmic nitrate reductase subunit (napA) gene, partial cds | 85 | 120 | 7.00E-19 |  |
| +NO_3_^-^ seq. 2 | HQ727721.1 | Uncultured Pseudomonas sp. clone Z-ALK-3 periplasmic nitrate reductase subunit (napA) gene, partial cds | 86.49 | 111 | 1.00E-20 |  |
| +NO_3_^-^ seq. 2 | HQ727722.1 | Uncultured bacterium clone Z-ALK-4 periplasmic nitrate reductase subunit (napA) gene, partial cds | 84.72 | 144 | 1.00E-23 |  |
| +NO_3_^-^ seq. 2 | Z46806.1 | Rhodobacter sphaeroides yntC, napK, napE, napF, napD, naB and napC genes | 82.35 | 119 | 4.00E-11 | *napB*  ( 2055-4550) |
| +NO_3_^-^ seq. 2 | AJ004933.1 | Rhodopseudomonas sp. napE, napD & napA genes of nap operon, 5' region | 82.35 | 204 | 1.00E-26 | *napA*  (739-2415) |
| +NO_3_^-^ seq. 2 | HE814004.1 | Pseudomonas stutzeri partial napA gene for periplasmic nitrate reductase large subunit precursor, strain Gr16 | 88.61 | 79 | 7.00E-16 |  |
| +NO_3_^-^ seq. 2 | HE814005.1 | Pseudomonas stutzeri partial napA gene for periplasmic nitrate reductase large subunit precursor, strain Gr17 | 87.34 | 79 | 2.00E-13 |  |
| +NO_3_^-^ seq. 2 | HE814006.1 | Pseudomonas stutzeri partial napA gene for periplasmic nitrate reductase large subunit precursor, strain Gr18 | 89.87 | 79 | 3.00E-18 |  |
| +NO_3_^-^ seq. 2 | HE814007.1 | Pseudomonas stutzeri partial napA gene for periplasmic nitrate reductase large subunit precursor, strain Gr19 | 88.61 | 79 | 7.00E-16 |  |
| +NO_3_^-^ seq. 2 | HE814008.1 | Pseudomonas stutzeri partial napA gene for periplasmic nitrate reductase large subunit precursor, strain Gr20 | 88.61 | 79 | 7.00E-16 |  |
| +NO_3_^-^ seq. 2 | HE814009.1 | Pseudomonas stutzeri partial napA gene for periplasmic nitrate reductase large subunit precursor, strain Gr21 | 86.08 | 79 | 3.00E-12 |  |
| +NO_3_^-^ seq. 2 | HE814010.1 | Pseudomonas stutzeri partial napA gene for periplasmic nitrate reductase large subunit precursor, strain Gr45 | 88.61 | 79 | 7.00E-16 |  |
| +NO_3_^-^ seq. 2 | HE814011.1 | Pseudomonas stutzeri partial napA gene for periplasmic nitrate reductase large subunit precursor, strain Gr46 | 87.34 | 79 | 2.00E-13 |  |
| +NO_3_^-^ seq. 2 | HE814012.1 | Pseudomonas stutzeri partial napA gene for periplasmic nitrate reductase large subunit precursor, strain Gr50 | 86.08 | 79 | 4.00E-11 |  |
| +NO_3_^-^ seq. 2 | HE814013.1 | Pseudomonas sp. Gr57 partial napA gene for periplasmic nitrate reductase large subunit precursor, strain Gr57 | 84.62 | 78 | 4.00E-08 |  |
| +NO_3_^-^ seq. 2 | HE814014.1 | Pseudomonas sp. Gr65 partial napA gene for periplasmic nitrate reductase large subunit precursor, strain Gr65 | 85.9 | 78 | 2.00E-10 |  |
| +NO_3_^-^ seq. 2 | X71385.1 | A.eutrophus genes napA and napB | 82.86 | 105 | 7.00E-10 | *napA*  (296=2791) |
| +NO_3_^-^ seq. 2 | AY515307.1 | Pseudomonas sp. Y2-1-1 periplasmic nitrate reductase NapA subunit (napA) gene, partial cds | 86.52 | 89 | 1.00E-14 |  |
| +NO_3_^-^ seq. 2 | DQ200356.1 | Pseudomonas stutzeri OrfX gene, partial cds; nap operon, complete sequence; and Dcd (dcd) gene, partial cds | 82.59 | 201 | 1.00E-26 | *napA*  (1159-3663) |
| +NO_3_^-^ seq. 2 | CP000146.1 | Rhodobacter sphaeroides 2.4.1 plasmid C, complete sequence | 83.19 | 119 | 2.00E-13 | *napA*  (80744-83239) |

^1^The query sequence indicates that only two sequences out of 28,688 in the +NO_3_^-^ metagenome matched with sequences in the N metabolism database. Seq. 1 matched with three database entries, while seq. 2 matched with 126 database entries.

^2^Gene matches specified only if they are unclear from the database match description. The region is the bp location on the database match for the gene.
